# Mental Health Promotion Among University Students Using Text Messaging: Protocol for a Randomized Controlled Trial of a Mobile Phone–Based Intervention

**DOI:** 10.2196/12396

**Published:** 2019-08-15

**Authors:** Kristin Thomas, Marcus Bendtsen

**Affiliations:** 1 Department of Medical and Health Sciences Faculty of Medicine Linköping University Linköping Sweden

**Keywords:** mental health, telemedicine, students, randomized controlled trial

## Abstract

**Background:**

There is a growing understanding that well-being and mental illness are 2 separate dimensions of mental health. High well-being is associated with decreased risk of disease and mental illness and increased longevity.

**Objective:**

This study aims to test the efficacy of a mobile phone–based intervention on positive mental health.

**Methods:**

We are conducting a 2-armed randomized controlled trial of university students in Sweden. Recruitment will last for 6 months by digital advertising (eg, university websites). Participants will be randomly allocated to either an intervention (fully automated mobile phone–based mental health intervention) or control group (treatment as usual). The primary outcome will be self-assessed positive mental health (Mental Health Continuum Short Form). Secondary outcomes will be self-assessed depression anxiety symptomatology (Hospital Anxiety Depression Scale). Outcomes will be investigated at baseline, at 3, 6, and 12 months after randomization. Mediators (positive emotions and thoughts) will be investigated at baseline, midintervention, and at follow-ups using 2 single face-valid items.

**Results:**

Data will be collected between autumn 2018 and spring 2019. Results are expected to be published in 2020.

**Conclusions:**

Strengths of the study include the use of a validated comprehensive instrument to measure positive mental health. Mechanisms of change are also investigated. A potential challenge could be recruitment; however, by setting a prolonged recruitment period, we believe that the study will recruit a sufficient sample.

**Trial Registration:**

International Standard Randomized Controlled Trial Number: 54748632; http://www.isrctn.com/ ISRCTN54748632

**International Registered Report Identifier (IRRID):**

PRR1-10.2196/12396

## Introduction

### Background

Mental well-being has traditionally been perceived as simply the absence of mental illness [1]. There is now evidence that positive mental health and mental illness are 2, albeit connected, unique dimensions of mental health. Positive experiences such as personal growth or feelings of hope can occur alongside mental illness. Similarly, the absence of mental illness does not mean automatic presence of positive mental health. Indeed, people afflicted by mental illness can have experiences of personal growth and feelings of hope, indicating that well-being and mental illness are not mutually exclusive [1-5].

Strong positive mental health is associated with various benefits including decreased risk of disease [6-10], decreased risk of mental illness [11-14], and increased longevity [8,15]. In a cohort study, individuals with poor positive mental health were 7 times more likely to be depressed 10 years later [14]. Individuals with poor positive mental health score similar to, or worse than, clinically depressed individuals on outcomes such as everyday functioning (eg, number of days absent from work) and psychosocial functioning (eg, achieving goals) [16]. The promotion of positive mental health among the general population has, therefore, recently been emphasized as the most important goal for the public mental health agenda in Europe [17].

Positive psychology interventions (PPIs) aim to promote positive mental health [18,19]. For example, a brief exercise where the individual is asked “to think about three things that went well during the day and savor those moments” have shown to increase positive mental health [9,19]. The positivity-activity model proposes that PPIs affect the relationship between positive practice (eg, acts of kindness) and well-being through increased positive emotions, thoughts, and behaviors. Furthermore, that features of positive practices (eg, how often do you perform acts of kindness) and individual factors (eg, motivation to be kind) moderate the relationship between positive practice and well-being [20].

Meta-analyses show that PPIs have a small effect on positive mental health in healthy volunteers and clinical populations [9,19]. However, 1 meta-analysis including 39 randomized controlled trials on the effect of PPIs showed that the quality of studies varied significantly, for example, blinding of participants, indicating a need for more rigorous studies. In addition, the majority of studies have investigated the effect of individual exercises targeting 1 aspect of positive mental health per se limiting the external validity of the results (eg, using a gratitude journal to increase positive thinking).

There is high acceptance among the general population to use mobile technology for health self-management, [21] and technical interventions can offer privacy and an emotionally safe environment [22]. Interventions using short message service (SMS) text messages are feasible to implement on a population level as nearly all people have a mobile phone; interventions could, therefore, reach a large proportion of a target group at low costs [23,24]. Thus, mobile phone–based interventions could be a cost-effective choice for mental health promotion and disseminating PPIs to larger audiences.

There is a great number of mobile apps commercially available aiming to promote mental health among the general population. A review of hundreds of these apps discovered that the majority lacked experimental evidence, were not theory-based, and had not been scientifically evaluated [25]. The research literature shows promising results whereby mobile phone–based interventions can increase well-being. A review on the effect of digital interventions (eg, mobile apps) on mental health showed a small to medium effect where mental health problems decreased and positive mental health increased. However, the overall quality of the studies was relatively low regarding risk of bias [26]. Another review summarized the evidence for theory-driven and evidence-based mental health electronic resources (e-resources; eg, website or mobile apps) and only found 1 randomized controlled trial. The authors concluded that e-resources for mental health have the potential to be widely effective; however, more rigorous studies that can clarify the evidence base are needed [27].

In addition, there is limited understanding on *how* PPIs work, that is, what mechanisms contribute to an increase in positive mental health? Although evidence suggests that PPIs elicit positive thoughts, emotions, and behaviors, which in turn increase well-being, few studies have investigated this proposition in the context of mobile phone interventions [20,28].

### Objectives

This protocol describes a randomized controlled trial that aims to test the efficacy of a fully automated mobile phone–based intervention on positive mental health among university students. The primary hypothesis is that participants in the intervention group will report significantly higher positive mental health (measured by the primary outcome measure) at follow-up compared with participants in the control group. Secondary hypotheses are that the intervention group will report significantly higher emotional, social, and psychological well-being as well as significantly lower rates of anxiety and depression symptomatology (measured by primary and secondary outcomes) at follow-ups compared with participants in the control group. These hypotheses are proposed at 3-, 6-, and 12-month follow-ups. Positive emotions and thoughts are hypothesized to mediate the relationship between access to the intervention and outcomes.

## Methods

### Design

A 2-arm randomized controlled trial will be conducted where participants will be equally allocated to either an intervention (mobile phone–based program) or control group (treatment as usual). No strata or blocks will be employed, and the randomization procedure will not be subverted as this and all subsequent study processes are fully automated.

### Outcomes and Measures

A baseline questionnaire will investigate demographic data (age, gender, and social status), primary outcome [2], and secondary outcomes [29]. Follow-up questionnaires will investigate primary and secondary outcomes. Outcomes will be investigated at baseline and at 3, 6, and 12 months after randomization. Participants will receive and complete questionnaires through their mobile phones.

Primary outcome will be positive mental health, assessed with the 14-item Mental Health Continuum Short Form (MHC-SF) [2]. Higher scores indicate greater emotional, social, and psychological well-being (range 0-84). Secondary outcomes will be depression and anxiety symptomatology assessed as the score on corresponding subscales of the Hospital Anxiety Depression Scale (HADS) [29].

To investigate mediators of the intervention [20], 2 face-valid items will measure the frequency of positive thoughts (‘‘During the last week, to what extent have you experienced positive thoughts” and “During the last week, to what extent have you experienced positive emotions”). Items will be rated on a scale ranging from 0= *not at all* to 9= *to a very high extent*. Mediators are assessed at baseline, midintervention (end of week 5), and at subsequent follow-ups. Mediation scores are hypothesized to be significantly higher in the intervention group compared with the control group.

### Participants and Inclusion and Exclusion Criteria

Inclusion criteria will be university students aged 18 to 29 years, able to read and understand Swedish, and owning a mobile phone. Exclusion criterion will be strong positive mental health defined as a score of 70 or more on the MHC-SF [2], as these individuals already demonstrate high positive mental health. A second exclusion criterion will be depression and anxiety symptomatology defined as a score of greater than or equal to 10 on both subscales of the HADS [29]. Owing to the proposed intervention not being a treatment program for HADS, these individuals will be given information about where to receive support.

### Intervention Group

The intervention is a fully automated mobile phone–based positive psychology multicomponent program. The content is based on the positive-activity model [20] and empirical evidence of PPIs from the positive psychology research field. The program runs for 10 weeks with a new theme being introduced each week. Each theme has shown to contribute to positive mental health in previous research: gratitude, savoring, positive emotions, personal strengths, positive relations, social environment, health behaviors, optimism, and goal setting (eg, [30-33]). During the last week, the user is guided to plan for the future and reflect on the program, for example, lessons learnt.

The program aims to increase users’ positive mental health and includes information about well-being, validated self-help exercises, tips, self-monitoring, and personalized feedback. The SMS text messages include text and links to pictures, interactive exercises, and further reading. SMS text messages are automatically sent to users throughout the program with on average 1 SMS text message a day.

### Control Group

Individuals allocated to the control setting will be informed of this through an SMS text message. The SMS text message will also include contact details of their local student health service, primary care center, or governmental national health website (treatment as usual).

### Sample Size

A power analysis was conducted to determine the necessary number of participants to invite to the study. To detect a standardized effect size of 0.3, so as to have the average score in the intervention group exceeding the scores of 62% of the control group, a total of 352 participants are required. The calculations were done assuming an 80% chance of detecting the difference at a significance level of .05 (2-tailed). Assuming that 70% of the participants respond to the follow-up questionnaire, it is necessary to recruit 503 participants in total. Furthermore, assuming that 1% of the invited population is willing to join the study (and are not excluded), we need to invite 50,300 participants.

### Recruitment

**Figure 1:**
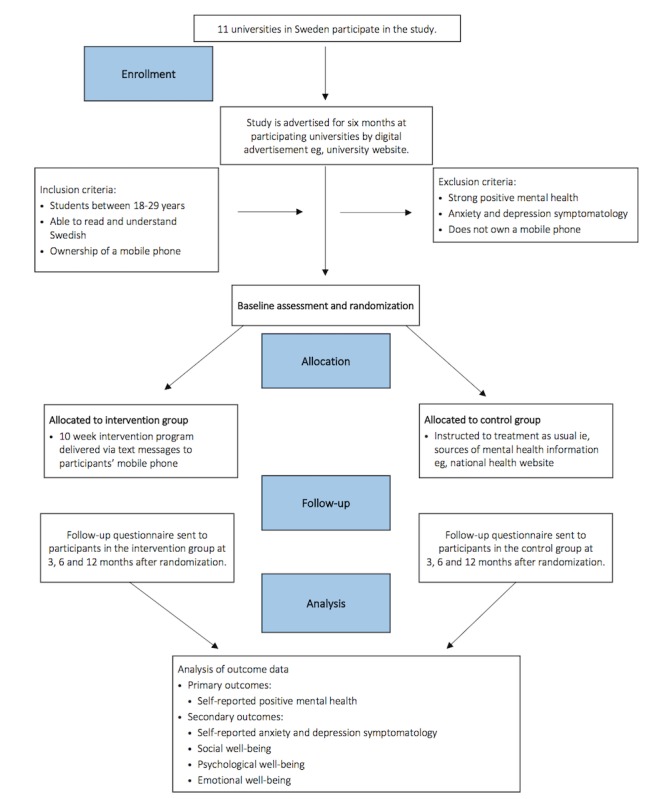
Flowchart of the recruitment procedure.

Students from 11 universities in Sweden will be invited to take part. Recruitment will last for 6 months and be executed by digital advertising (email, university websites, Student Health Services websites, and learning management systems used by the universities). The universities are located throughout Sweden in rural and urban settings. Faculties from medical, technical, art, and social sciences will be represented. The advertisement will include information on the study aims, confidentiality, and trial design. Students will register their interest by sending an SMS text message to a dedicated telephone number (included in the advertisement material). Informed consent and the baseline questionnaire will be completed on their mobile phone. After completing the baseline questionnaire, students will then automatically be randomized to either an intervention or control group. Participants will know that they have been randomized to either an intervention or control group. Figure 1 depicts a flowchart of the recruitment procedure of the study.

### Randomization

Participants will be randomized to either the intervention or control group. Each participant will be allocated a number 1 or 2 with equal probabilities using Java’s built-in random number generator (java.util.Random). Randomization is thus fully computerized, does not use any strata or blocks, and is not possible to subvert because this and all subsequent study processes are fully automated.

### Statistical Methods

All analyses will be done under the intention-to-treat principle, where all randomized individuals will be included. Missing outcome data will initially be handled by a complete-case analysis, which assumes that data are missing at random. If data are systematically missing, then it may be the case that early responders differ from late responders, and in extension that late responders are more similar to nonresponders. We will, therefore, explore the plausibility of the missing at random assumption by regressing the primary outcomes on the number of follow-up attempts needed before a response was recorded. To further explore the missing at random assumption, attrition will be investigated among study groups by comparing baseline characteristics between those who did and did not respond at follow-up.

For all models, coefficients of interest will be assessed for statistical significance using a null hypothesis testing approach, where tests will be 2-tailed at the .05 significance level. Alongside the null hypothesis tests, posterior distributions using a Bayesian approach will be calculated for each coefficient. Both significance tests and posterior distributions will create a basis for scientific inference [34].

The primary outcome (MHC-SF total score) is a discrete measure, which may be skewed; thus, we will regress this outcome on group allocation using negative binomial regression. Both unadjusted and adjusted models will be explored (adjusting for demographics, total MHC-SF, and mediator variables at baseline).

The 3 subscales of MHC-SF (emotional well-being, social well-being, and psychological well-being) are mean scores from Likert scale items, which should, because of the law of large numbers, tend toward normality. If by visual inspection it is deemed that the measures are skewed, we will use log transformation. We will regress the individual scores against group allocation using normal linear regression. Both unadjusted and adjusted models will be explored (adjusting for demographics and each score, respectively, and mediator variables at baseline).

Depression and anxiety scores from HADS will also be regressed against group allocation using negative binomial regression. Both unadjusted and adjusted models will be explored (adjusting for demographics, depression, and anxiety, respectively, and mediator variables at baseline).

Mediators will be explored using a causal inference framework, where Monte Carlo methods are relied upon for inference. A total of 3 models will be created for each outcome measure: 2 that investigate the mediating factors on their own and a third model that incorporates all mediators at once.

Effect modification tests will be performed in all models to assess if any of the baseline characteristics moderate the effect of the intervention. Adjusted models will be primary.

### Ethical Statement

The study has received ethical approval from the Regional Ethical Review Board, Linköping University, Sweden (Dnr 2018/519-32).

## Results

Data will be collected between autumn 2018 and spring 2019. Results are expected to be published in 2020.

## Discussion

### Overview

This paper describes the design of a study that will evaluate the effect of a mobile phone–based intervention to increase positive mental health among university students. This study will add knowledge to the efficacy of a fully automated PPI. Previous research has investigated partly digital interventions and often only included single component interventions.

### Strengths and Limitations

A potential challenge could be recruiting sufficient number of participants. According to our power analysis, we need just above 500 participants to achieve 80% power. By including some of the larger universities in Sweden and setting the recruitment period to 6 months, we believe that the study will recruit a sufficient sample.

A strength of the study is that it will investigate potential mechanisms of change. The proposed mediators in the study (positive emotions and cognitions) are based on a framework of mechanisms of positive interventions (the positivity-activity model). However, the challenge is how to measure these mediators in a reliable and feasible way. In a randomized controlled trial with already comprehensive baseline and follow-up questionnaires, it was not realistic to include lengthy validated instruments of, for example, positive emotions. A total of 2 single face valid items were therefore used to measure proposed mediators.

Another strength of the study is the use of the MHC-SF [2] to measure positive mental health. It is a validated comprehensive instrument including emotional and social well-being as well as psychological function. Positive mental health is a complex construct that requires the use of an instrument that captures both the hedonic and eudaimonic dimensions of well-being.

### Conclusions

The promotion of positive mental health among the general population is increasingly becoming a core component of the public mental health agenda in Europe [17]. Mobile phone–based interventions could be a cost-effective choice for mental health promotion and an effective way to disseminate evidence-based interventions to larger audiences. However, more rigorous and larger studies that can clarify the evidence base in this area are needed [27]. This protocol describes a randomized controlled trial investigating a newly developed, fully automated, mobile phone–based intervention promoting well-being among university students. The trial can contribute to the knowledge on the feasibility and effect of the mobile phone–based intervention in promoting mental health.

## Abbreviations

e-resource: electronic resource
HADS: Hospital Anxiety Depression Scale
MHC-SF: Mental Health Continuum Short Form
PPI: positive psychology intervention
SMS: short message service
